# The genome sequence of the Currant Clearwing, *Synanthedon tipuliformis *(Clerck, 1759)

**DOI:** 10.12688/wellcomeopenres.19647.1

**Published:** 2023-07-11

**Authors:** Douglas Boyes, Peter W. H. Holland, University of Oxford and Wytham Woods Genome Acquisition Lab, Darwin Tree of Life Barcoding collective, Wellcome Sanger Institute Tree of Life programme, Wellcome Sanger Institute Scientific Operations: DNA Pipelines collective, Tree of Life Core Informatics collective, Darwin Tree of Life Consortium

**Affiliations:** 1 UK Centre for Ecology & Hydrology, Wallingford, England, UK; 2 University of Oxford, Oxford, England, UK

**Keywords:** Synanthedon tipuliformis, Currant Clearwing, genome sequence, chromosomal, Lepidoptera

## Abstract

We present a genome assembly from an individual male *Synanthedon tipuliformis* (the Currant Clearwing; Arthropoda; Insecta; Lepidoptera; Sesiidae). The genome sequence is 295.8 megabases in span. Most of the assembly is scaffolded into 31 chromosomal pseudomolecules, including the Z sex chromosome. The mitochondrial genome has also been assembled and is 27.05 kilobases in length.

## Species taxonomy

Eukaryota; Metazoa; Eumetazoa; Bilateria; Protostomia; Ecdysozoa; Panarthropoda; Arthropoda; Mandibulata; Pancrustacea; Hexapoda; Insecta; Dicondylia; Pterygota; Neoptera; Endopterygota; Amphiesmenoptera; Lepidoptera; Glossata; Neolepidoptera; Heteroneura; Ditrysia; Apoditrysia; Sesioidea; Sesiidae; Sesiinae; Synanthedonini; *Synanthedon*; *Synanthedon tipuliformis* (Clerck, 1759) (NCBI:txid301680).

## Background

The family Sesiidae contains over 1500 species of diurnal moths, most of which have transparent wing regions devoid of scales, hence their common name ‘clearwings’. Many have yellow, black or red markings on the body and are thought to be Batesian mimics of wasps and bees. The Currant Clearwing or Currant Borer, *Synanthedon tipuliformis*, is found across Europe and further east in Eurasia through Ukraine, Georgia, Belarus and Russia ( GBIF Secretariat, 2022). The moth has been recorded across much of England and Wales but is rare in Scotland and Northern Ireland; it is also rare in Ireland ( MothsIreland, 2023; Randle *et al.*, 2019). The species has spread outside its natural range as an accidentally introduced species in many countries including Australia, New Zealand, the United States and Canada ( GBIF Secretariat, 2022).

The adult moth is diurnal and is on the wing in summer and can be seen settling on currant plants ( *Ribes* sp.) on sunny days; in southern England the peak flight period spans June and July. Males are also readily attracted to pheromone lures. The female sex pheromone was identified as a blend of ~98% E,Z-2,13-octadecadienyl acetate and ~2% E,Z-3,13-octadecadienyl acetate; this mix has proved an effective attractant in Europe, New Zealand, Canada and the United States. A study suggesting a slightly different pheromone mix in Tasmania was later disputed ( James *et al.*, 2001; Suckling *et al.*, 2005). Females lay eggs on the buds or bark of the larval foodplant, usually blackcurrant ( *Ribes nigrum*) or redcurrant ( *Ribes rubrum*). Larvae burrow into the stems where they feed internally throughout summer and autumn before overwintering and completing larval development in spring ( Grassi *et al.*, 2002). The internal feeding weakens the plants and impacts fruit yield, and consequently the species can become a pest on commercial currant farms. Pheromones have been used successfully to disrupt mating and reduce numbers of the moth when population densities are high ( Suckling *et al.*, 2005). An alternative control method, using a parasitic nematode, *Neoaplectanabibionis*, has been attempted in Tasmania ( Miller & Bedding, 1982).

The complete genome of *Synanthedon tipuliformis* was determined as part of the Darwin Tree of Life project. The assembled genome will facilitate research into pest control strategies and contribute to the growing set of resources for studying insect ecology and evolution.

## Genome sequence report

**Figure 1.**
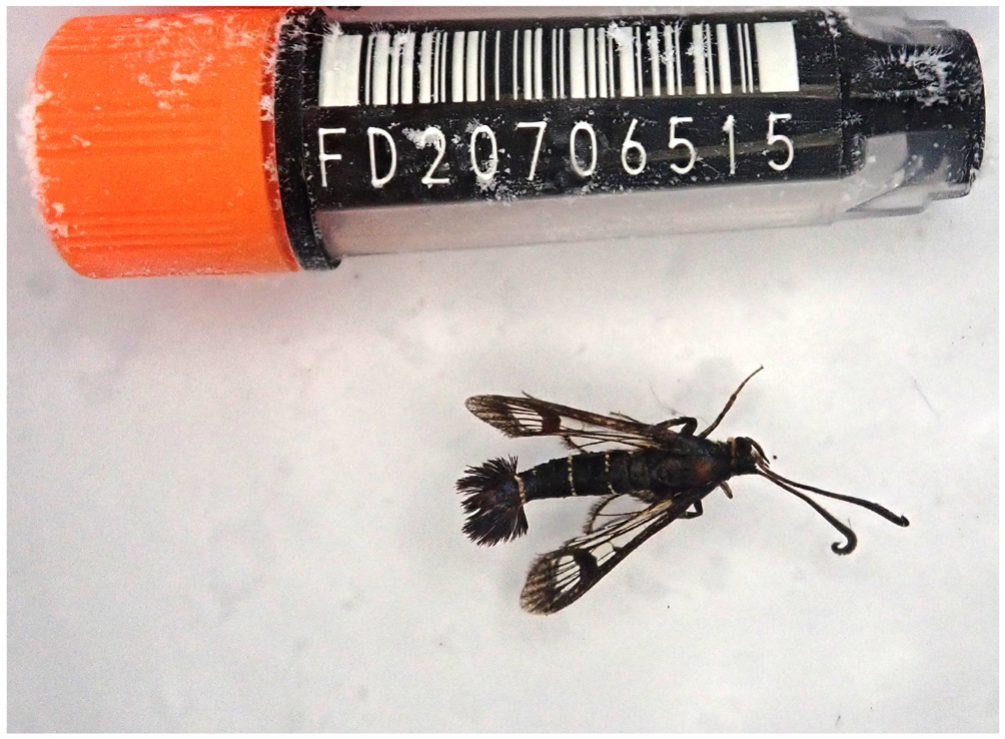
Photograph of the *Synanthedon tipuliformis* (ilSynTipu2) specimen used for genome sequencing.

The genome was sequenced from one male *Synanthedon tipuliformis* ( Figure 1) collected from Wytham Woods, Oxfordshire, UK (51.77, –1.31). A total of 66-fold coverage in Pacific Biosciences single-molecule HiFi long reads was generated. Primary assembly contigs were scaffolded with chromosome conformation Hi-C data. Manual assembly curation corrected 5 missingjoins or mis-joins.

**Figure 2.**
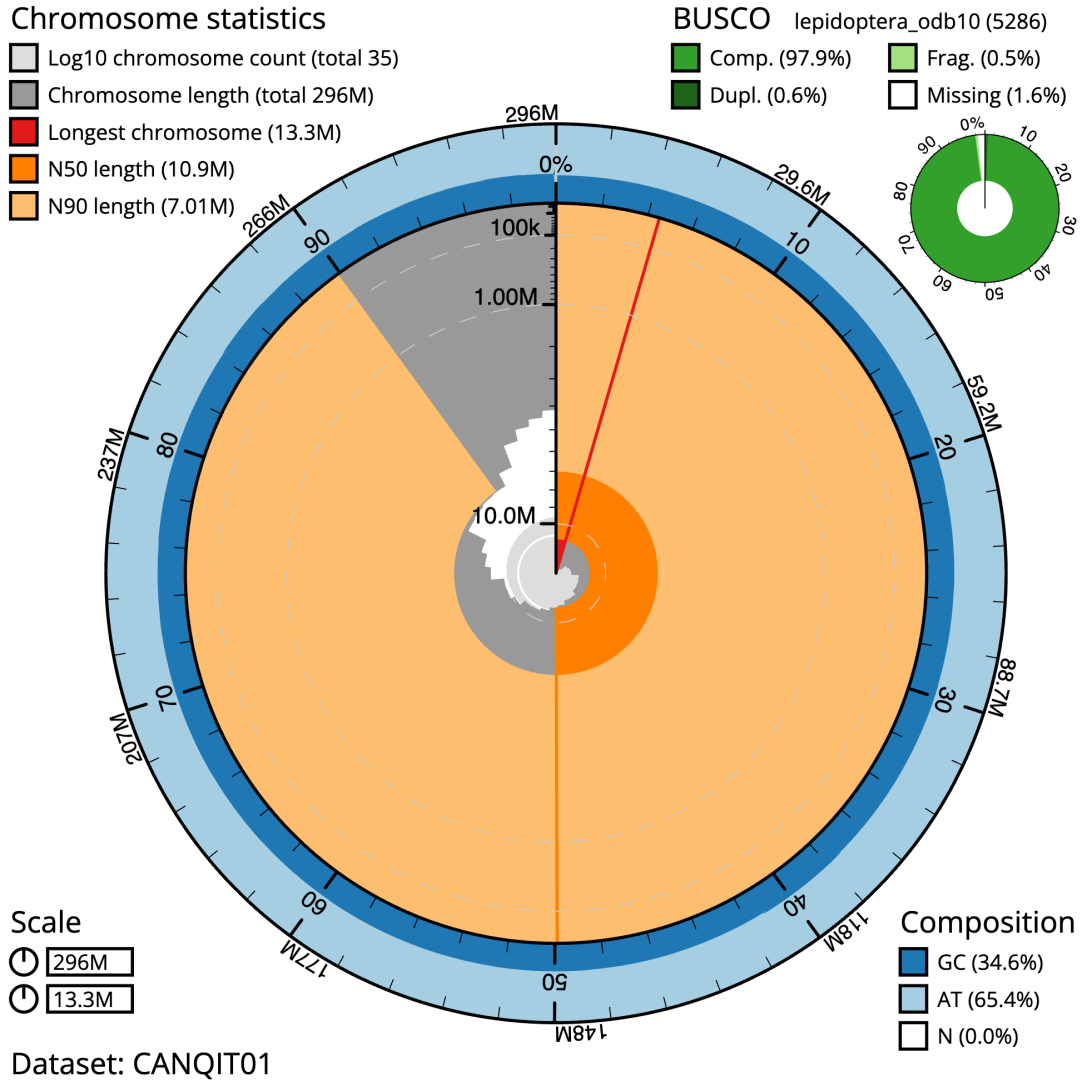
Genome assembly of *Synanthedon tipuliformis*, ilSynTipu2.1: metrics. The BlobToolKit Snailplot shows N50 metrics and BUSCO gene completeness. The main plot is divided into 1,000 size-ordered bins around the circumference with each bin representing 0.1% of the 295,831,181 bp assembly. The distribution of scaffold lengths is shown in dark grey with the plot radius scaled to the longest scaffold present in the assembly (13,326,013 bp, shown in red). Orange and pale-orange arcs show the N50 and N90 scaffold lengths (10,885,013 and 7,008,013 bp), respectively. The pale grey spiral shows the cumulative scaffold count on a log scale with white scale lines showing successive orders of magnitude. The blue and pale-blue area around the outside of the plot shows the distribution of GC, AT and N percentages in the same bins as the inner plot. A summary of complete, fragmented, duplicated and missing BUSCO genes in the lepidoptera_odb10 set is shown in the top right. An interactive version of this figure is available at https://blobtoolkit.genomehubs.org/view/ilSynTipu2.1/dataset/CANQIT01/snail.

The final assembly has a total length of 295.8 Mb in 34 sequence scaffolds with a scaffold N50 of 10.9 Mb ( Table 1). Most (99.98%) of the assembly sequence was assigned to 31 chromosomal-level scaffolds, representing 30 autosomes and the Z sex chromosome. Chromosome-scale scaffolds confirmed by the Hi-C data are named in order of size ( Figure 2– Figure 5; Table 2). The Z chromosome was identified based on alignment with that of *Synanthedon formicaeformis* (ilSynForm1; GCA_945859745.1). While not fully phased, the assembly deposited is of one haplotype. Contigs corresponding to the second haplotype have also been deposited. The mitochondrial genome was also assembled and can be found as a contig within the multifasta file of the genome submission.

**Table 1.** Genome data for *Synanthedon tipuliformis*, ilSynTipu2.1.

Project accession data		
Assembly identifier	ilSynTipu2.1	
Species	*Synanthedon tipuliformis*	
Specimen	ilSynTipu2	
NCBI taxonomy ID	301680	
BioProject	PRJEB57662	
BioSample ID	SAMEA10978931	
Isolate information	ilSynTipu2, male: head and thorax (DNA sequencing and Hi-C scaffolding)	
Assembly metrics *		*Benchmark*
Consensus quality (QV)	62.8	*≥ 50*
*k*-mer completeness	100%	*≥ 95%*
BUSCO **	C:97.9%[S:97.3%,D:0.6%], F:0.5%,M:1.6%,n:5,286	*C ≥ 95%*
Percentage of assembly mapped to chromosomes	99.98%	*≥ 95%*
Sex chromosomes	Z chromosome	*localised homologous pairs*
Organelles	Mitochondrial genome assembled	*complete single alleles*
Raw data accessions		
PacificBiosciences SEQUEL II	ERR10499353	
Hi-C Illumina	ERR10501011	
Genome assembly		
Assembly accession	GCA_947623395.1	
*Accession of alternate haplotype*	GCA_947623135.1	
Span (Mb)	295.8	
Number of contigs	61	
Contig N50 length (Mb)	9.8	
Number of scaffolds	34	
Scaffold N50 length (Mb)	10.9	
Longest scaffold (Mb)	13.33	

* Assembly metric benchmarks are adapted from column VGP-2020 of “Table 1: Proposed standards and metrics for defining genome assembly quality” from ( Rhie *et al.*, 2021). ** BUSCO scores based on the lepidoptera_odb10 BUSCO set using v5.3.2. C = complete [S = single copy, D = duplicated], F = fragmented, M = missing, n = number of orthologues in comparison. A full set of BUSCO scores is available at https://blobtoolkit.genomehubs.org/view/ilSynTipu2.1/dataset/CANQIT01/busco.

**Figure 3.**
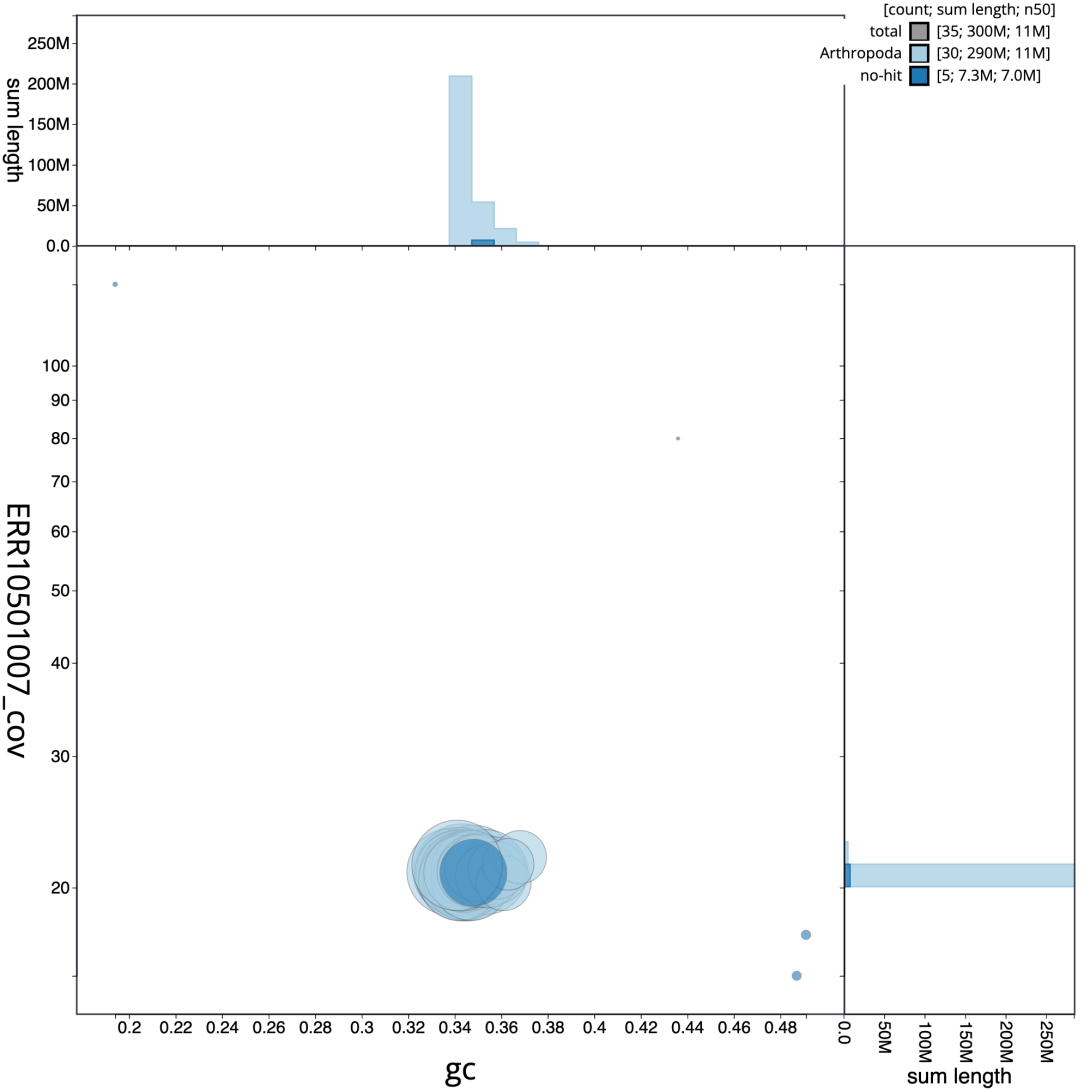
Genome assembly of *Synanthedon tipuliformis*, ilSynTipu2.1: BlobToolKit GC-coverage plot. Scaffolds are coloured by phylum. Circles are sized in proportion to scaffold length. Histograms show the distribution of scaffold length sum along each axis. An interactive version of this figure is available at https://blobtoolkit.genomehubs.org/view/ilSynTipu2.1/dataset/CANQIT01/blob.

**Figure 4.**
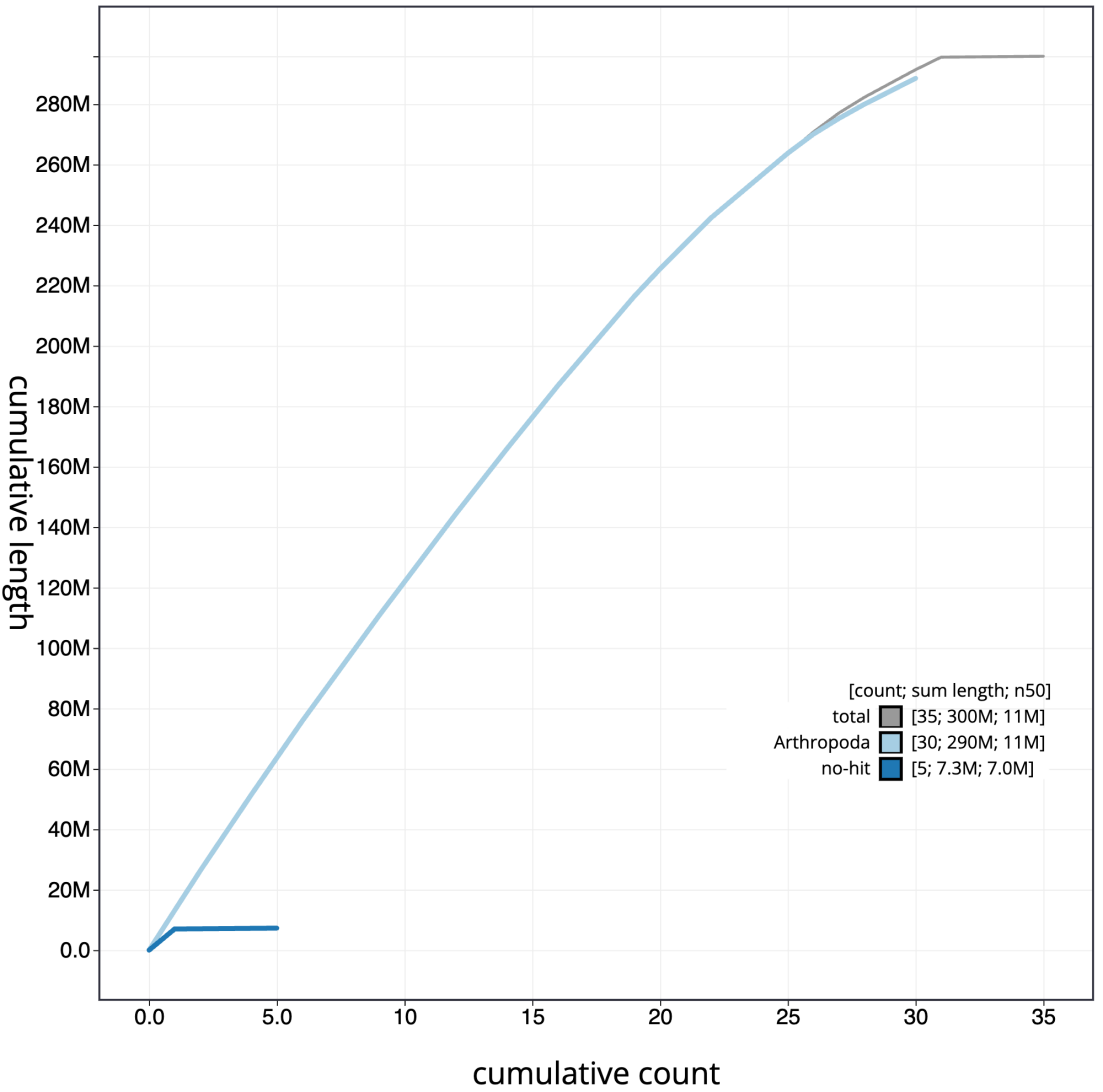
Genome assembly of *Synanthedon tipuliformis*, ilSynTipu2.1: BlobToolKit cumulative sequence plot. The grey line shows cumulative length for all scaffolds. Coloured lines show cumulative lengths of scaffolds assigned to each phylum using the buscogenestaxrule. An interactive version of this figure is available at https://blobtoolkit.genomehubs.org/view/ilSynTipu2.1/dataset/CANQIT01/cumulative.

**Figure 5.**
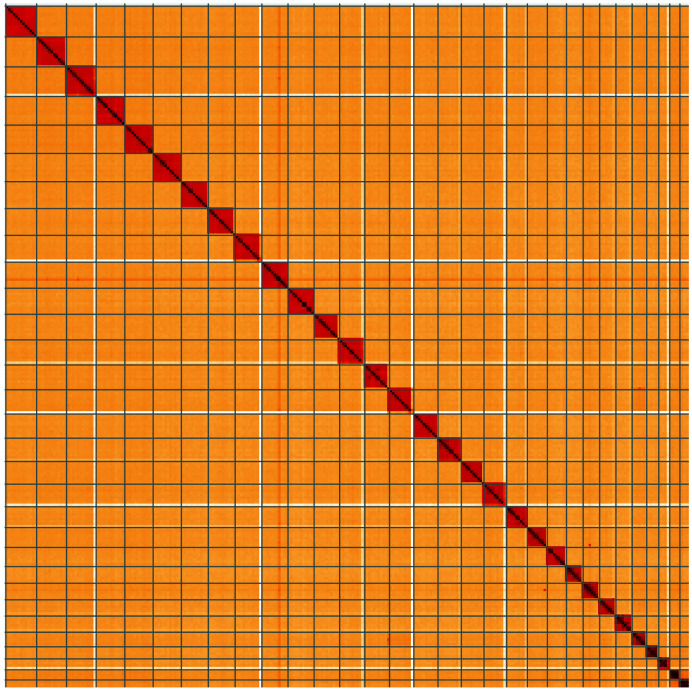
Genome assembly of *Synanthedon tipuliformis*, ilSynTipu2.1: Hi-C contact map of the ilSynTipu2.1 assembly, visualised using HiGlass. Chromosomes are shown in order of size from left to right and top to bottom. An interactive version of this figure may be viewed at https://genome-note-higlass.tol.sanger.ac.uk/l/?d=El18riT_TCymeEyYk8GCgQ.

**Table 2.** Chromosomal pseudomolecules in the genome assembly of *Synanthedon tipuliformis*, ilSynTipu2.

INSDC accession	Chromosome	Length (Mb)	GC%
OX392407.1	1	13.33	34.5
OX392408.1	2	12.93	34.5
OX392410.1	3	12.39	34.5
OX392411.1	4	12.26	35.0
OX392412.1	5	12.18	34.0
OX392413.1	6	11.66	34.0
OX392414.1	7	11.6	34.0
OX392415.1	8	11.56	34.5
OX392416.1	9	11.29	35.5
OX392417.1	10	11.28	34.0
OX392418.1	11	11.04	34.5
OX392419.1	12	10.89	34.5
OX392420.1	13	10.68	34.5
OX392421.1	14	10.5	34.5
OX392422.1	15	10.46	34.0
OX392423.1	16	10.06	34.5
OX392424.1	17	9.82	34.5
OX392425.1	18	9.78	34.5
OX392426.1	19	8.99	34.5
OX392427.1	20	8.63	35.0
OX392428.1	21	8.28	35.0
OX392429.1	22	7.19	34.5
OX392430.1	23	7.13	35.5
OX392431.1	24	7.05	35.0
OX392432.1	25	7.01	35.0
OX392433.1	26	6.3	35.5
OX392434.1	27	5.21	36.0
OX392435.1	28	4.71	36.0
OX392436.1	29	4.41	37.0
OX392437.1	30	4.13	36.5
OX392409.1	Z	12.81	34.0
OX392438.1	MT	0.03	19.5

The estimated Quality Value (QV) of the final assembly is 62.8 with *k*-mer completeness of 100%, and the assembly has a BUSCO v5.3.2 completeness of 97.9% (single =97.3%, duplicated = 0.6%), using the lepidoptera_odb10 reference set ( *n* = 5,286).

Metadata for specimens, spectral estimates, sequencing runs, contaminants and pre-curation assembly statistics can be found at https://links.tol.sanger.ac.uk/species/301680.

## Methods

### Sample acquisition and nucleic acid extraction

Amale *Synanthedon tipuliformis* (specimen ID Ox001662, individual ilSynTipu2) was collected using a pheromone lure in Wytham Woods, Oxfordshire, UK (latitude 51.77, longitude –1.31) on 2021-07-17. The specimen was collected and identified by Douglas Boyes (University of Oxford) and preserved on dry ice.

The ilSynTipu2 sample was prepared for DNA extraction at the Tree of Life laboratory, Wellcome Sanger Institute (WSI). The ilSynTipu2 sample was weighed and dissected on dry ice with tissue set aside for Hi-C sequencing. Head and thorax tissue was disrupted using a Nippi Powermasher fitted with a BioMasher pestle. DNA was extracted at the WSI Scientific Operations core using the Qiagen MagAttract HMW DNA kit, according to the manufacturer’s instructions.

### Sequencing

Pacific Biosciences HiFi circular consensus DNA sequencing libraries were constructed according to the manufacturers’ instructions. DNA sequencing was performed by the Scientific Operations core at the WSI on a Pacific Biosciences SEQUEL II (HiFi) instrument. Hi-C data were also generated from head tissue of ilSynTipu2 using the Arima2 kit and sequenced on the Illumina NovaSeq 6000 instrument.

### Genome assembly, curation and evaluation

Assembly was carried out with Hifiasm ( Cheng *et al.*, 2021) and haplotypic duplication was identified and removed with purge_dups ( Guan *et al.*, 2020). The assembly was then scaffolded with Hi-C data ( Rao *et al.*, 2014) using YaHS ( Zhou *et al.*, 2023). The assembly was checked for contamination and corrected using the gEVAL system ( Chow *et al.*, 2016) as described previously ( Howe *et al.*, 2021). Manual curation was performed using gEVAL, HiGlass ( Kerpedjiev *et al.*, 2018) and Pretext ( Harry, 2022). The mitochondrial genome was assembled using MitoHiFi ( Uliano-Silva *et al.*, 2022), which runs MitoFinder ( Allio *et al.*, 2020) or MITOS ( Bernt *et al.*, 2013) and uses these annotations to select the final mitochondrial contig and to ensure the general quality of the sequence.

A Hi-C map for the final assembly was produced using bwa-mem2 ( Vasimuddin *et al.*, 2019) in the Cooler file format ( Abdennur & Mirny, 2020). To assess the assembly metrics, the *k*-mer completeness and QV consensus quality values were calculated in Merqury ( Rhie *et al.*, 2020). This work was done using Nextflow ( Di Tommaso *et al.*, 2017) DSL2 pipelines “sanger-tol/readmapping” ( Surana *et al.*, 2023a) and “sanger-tol/genomenote” ( Surana *et al.*, 2023b). The genome was analysed within the BlobToolKit environment ( Challis *et al.*, 2020) and BUSCO scores ( Manni *et al.*, 2021; Simão *et al.*, 2015) were calculated.

Table 3 contains a list of relevant software tool versions and sources.

**Table 3.** Software tools: versions and sources.

Software tool	Version	Source
BlobToolKit	4.1.7	https://github.com/blobtoolkit/blobtoolkit
BUSCO	5.3.2	https://gitlab.com/ezlab/busco
gEVAL	N/A	https://geval.org.uk/
Hifiasm	0.16.1-r375	https://github.com/chhylp123/hifiasm
HiGlass	1.11.6	https://github.com/higlass/higlass
Merqury	MerquryFK	https://github.com/thegenemyers/MERQURY.FK
MitoHiFi	2	https://github.com/marcelauliano/MitoHiFi
PretextView	0.2	https://github.com/wtsi-hpag/PretextView
purge_dups	1.2.3	https://github.com/dfguan/purge_dups
sanger-tol/genomenote	v1.0	https://github.com/sanger-tol/genomenote
sanger-tol/readmapping	1.1.0	https://github.com/sanger-tol/readmapping/tree/1.1.0
YaHS	yahs-1.1.91eebc2	https://github.com/c-zhou/yahs

### Wellcome Sanger Institute – Legal and Governance

The materials that have contributed to this genome note have been supplied by a Darwin Tree of Life Partner. The submission of materials by a Darwin Tree of Life Partner is subject to the **‘Darwin Tree of Life Project Sampling Code of Practice’**, which can be found in full on the Darwin Tree of Life website here. By agreeing with and signing up to the Sampling Code of Practice, the Darwin Tree of Life Partner agrees they will meet the legal and ethical requirements and standards set out within this document in respect of all samples acquired for, and supplied to, the Darwin Tree of Life Project.

Further, the Wellcome Sanger Institute employs a process whereby due diligence is carried out proportionate to the nature of the materials themselves, and the circumstances under which they have been/are to be collected and provided for use. The purpose of this is to address and mitigate any potential legal and/or ethical implications of receipt and use of the materials as part of the research project, and to ensure that in doing so we align with best practice wherever possible. The overarching areas of consideration are:

- Ethical review of provenance and sourcing of the material
- Legality of collection, transfer and use (national and international)

Each transfer of samples is further undertaken according to a Research Collaboration Agreement or Material Transfer Agreement entered into by the Darwin Tree of Life Partner, Genome Research Limited (operating as the Wellcome Sanger Institute), and in some circumstances other Darwin Tree of Life collaborators.

## Data Availability

European Nucleotide Archive: *Synanthedon tipuliformis* (currant clearwing). Accession number PRJEB57662; https://identifiers.org/ena.embl/PRJEB57662. ( Wellcome Sanger Institute, 2023) The genome sequence is released openly for reuse. The *Synanthedon tipuliformis* genome sequencing initiative is part of the Darwin Tree of Life (DToL) project. All raw sequence data and the assembly have been deposited in INSDC databases. The genome will be annotated using available RNA-Seq data and presented through the Ensembl pipeline at the European Bioinformatics Institute. Raw data and assembly accession identifiers are reported in Table 1. Members of the University of Oxford and Wytham Woods Genome Acquisition Lab are listed here: https://doi.org/10.5281/zenodo.4789928. Members of the Darwin Tree of Life Barcoding collective are listed here: https://doi.org/10.5281/zenodo.4893703. Members of the Wellcome Sanger Institute Tree of Life programme are listed here: https://doi.org/10.5281/zenodo.4783585. Members of Wellcome Sanger Institute Scientific Operations: DNA Pipelines collective are listed here: https://doi.org/10.5281/zenodo.4790455. Members of the Tree of Life Core Informatics collective are listed here: https://doi.org/10.5281/zenodo.5013541. Members of the Darwin Tree of Life Consortium are listed here: https://doi.org/10.5281/zenodo.4783558.
