# Supplementary material for: The genome sequence of the Currant Clearwing moth, Synanthedon tipuliformis (Clerck, 1759)
Source: Wellcome Open Res. 2024 Oct 11;8:300. Originally published 2023 Jul 11. [Version 2] doi: 10.12688/wellcomeopenres.19647.2 (PMC11499843; doi:10.12688/wellcomeopenres.19647.2)
Supplement: Peer review history

# Reviewer report: Reviewer response for version 2

**DOI:** 10.21956/wellcomeopenres.25594.r104608

**Published:** 2024-10-23

**Authors:** Josephine Paris

**Affiliations:** 1 DISVA, Marche Polytechnic University, Ancona, Ancona, Italy

I have checked the new version of the Data Note and can confirm that I approve with the revisions made.

Are sufficient details of methods and materials provided to allow replication by others?

Partly

Is the rationale for creating the dataset(s) clearly described?

Yes

Are the datasets clearly presented in a useable and accessible format?

Yes

Are the protocols appropriate and is the work technically sound?

Yes

Reviewer Expertise:

population genomics of non-model species, genome assembly and annotation

I confirm that I have read this submission and believe that I have an appropriate level of expertise to confirm that it is of an acceptable scientific standard.

# Reviewer report: Reviewer response for version 2

**DOI:** 10.21956/wellcomeopenres.25594.r104607

**Published:** 2024-10-16

**Authors:** Annabel Whibley

**Affiliations:** 1 The University of Auckland, Auckland, Auckland, New Zealand; 2 Bragato Research Institute, Lincoln, New Zealand

No additional comments.

Are sufficient details of methods and materials provided to allow replication by others?

Yes

Is the rationale for creating the dataset(s) clearly described?

Yes

Are the datasets clearly presented in a useable and accessible format?

Yes

Are the protocols appropriate and is the work technically sound?

Yes

Reviewer Expertise:

Genomics, Bioinformatics, Evolution

I confirm that I have read this submission and believe that I have an appropriate level of expertise to confirm that it is of an acceptable scientific standard.

# Reviewer report: Reviewer response for version 1

**DOI:** 10.21956/wellcomeopenres.21764.r87436

**Published:** 2024-07-31

**Authors:** Annabel Whibley

**Affiliations:** 1 The University of Auckland, Auckland, Auckland, New Zealand; 2 Bragato Research Institute, Lincoln, New Zealand

In this Data Note, Boyes, Holland and colleagues present a reference genome assembly of the Currant Clearwing ( *Synanthedon tipuliformis).* An annotation has not been developed at this time. The natural history background is engaging and notes interesting features about the moth and its control in locations where it is considered a pest. The assembly is of high-quality using appropriate methods and with comprehensive reporting of lab and bioinformatics methods and metadata. Links to data accessions are all working.

**Minor comments:**

 Please preface geographical co-ordinates with latitude and longitude.

 There is a missing space (“missingjoins”) in the last sentence of the Genome sequence report  introduction, before Figure 1.

 Also a missing space (“Amale”)  in the first sentence of the Methods.

Are sufficient details of methods and materials provided to allow replication by others?

Yes

Is the rationale for creating the dataset(s) clearly described?

Yes

Are the datasets clearly presented in a useable and accessible format?

Yes

Are the protocols appropriate and is the work technically sound?

Yes

Reviewer Expertise:

Genomics, Bioinformatics, Evolution

I confirm that I have read this submission and believe that I have an appropriate level of expertise to confirm that it is of an acceptable scientific standard.

# Reviewer report: Reviewer response for version 1

**DOI:** 10.21956/wellcomeopenres.21764.r84922

**Published:** 2024-06-14

**Authors:** Josephine Paris

**Affiliations:** 1 DISVA, Marche Polytechnic University, Ancona, Ancona, Italy

This Data Note reports a chromosome-level genome assembly for the Currant Clearwing, a diurnal moth species which is invasive outside of Europe, and is considered a pest to currant plants. The Background section includes a clear introduction to the biology of the species and highlights the use of the genome for future work. The genome assembly is of very good quality, and an annotation will be performed using RNAseq data at a later date by EBI. The genome is presented with the standard quality metrics for a genome assembly published as a Data Note, and most of the information for replication is present. I have no major comments, and just five minor comments:

 - Please include in the Abstract how much of the assembly was scaffolded, rather than "Most"

 - Please include how much Hi-C data was generated for scaffolding

 - Please include if MITOS or MitoFinder was used for the mitogenome assembly

 - A space is missing in "missingjoins"

 - The first sentence of the methods says "Amale" - a space is missing here

 Finally, I have checked the availability of the data on the ENA and everything is present and accessible.

Are sufficient details of methods and materials provided to allow replication by others?

Partly

Is the rationale for creating the dataset(s) clearly described?

Yes

Are the datasets clearly presented in a useable and accessible format?

Yes

Are the protocols appropriate and is the work technically sound?

Yes

Reviewer Expertise:

population genomics of non-model species, genome assembly and annotation

I confirm that I have read this submission and believe that I have an appropriate level of expertise to confirm that it is of an acceptable scientific standard, however I have significant reservations, as outlined above.
